# The Impact of Natural Compounds on S-Shaped Aβ42 Fibril: From Molecular Docking to Biophysical Characterization

**DOI:** 10.3390/ijms21062017

**Published:** 2020-03-16

**Authors:** Stefano Muscat, Lorenzo Pallante, Filip Stojceski, Andrea Danani, Gianvito Grasso, Marco Agostino Deriu

**Affiliations:** 1 Dalle Molle Institute for Artificial Intelligence (IDSIA), University of Italian Switzerland (USI), University of Applied Science and Art of Southern Switzerland (SUPSI), CH-6928 Manno, Switzerland; 2 PolitoBIOMed Lab, Department of Mechanical and Aerospace Engineering, Politecnico di Torino, IT-10128 Torino, Italy

**Keywords:** Alzheimer’s disease, Amyloid β, natural compounds, molecular dynamics, ensemble docking, S-shape

## Abstract

The pursuit for effective strategies inhibiting the amyloidogenic process in neurodegenerative disorders, such as Alzheimer’s disease (AD), remains one of the main unsolved issues, and only a few drugs have demonstrated to delay the degeneration of the cognitive system. Moreover, most therapies induce severe side effects and are not effective at all stages of the illness. The need to find novel and reliable drugs appears therefore of primary importance. In this context, natural compounds have shown interesting beneficial effects on the onset and progression of neurodegenerative diseases, exhibiting a great inhibitory activity on the formation of amyloid aggregates and proving to be effective in many preclinical and clinical studies. However, their inhibitory mechanism is still unclear. In this work, ensemble docking and molecular dynamics simulations on S-shaped Aβ_42_ fibrils have been carried out to evaluate the influence of several natural compounds on amyloid conformational behaviour. A deep understanding of the interaction mechanisms between natural compounds and Aβ aggregates may play a key role to pave the way for design, discovery and optimization strategies toward an efficient destabilization of toxic amyloid assemblies.

## 1. Introduction

Alzheimer disease (AD) is one of the most common forms of dementia. The mechanism of Alzheimer’s onset and progression is still unclear, and several hypotheses have been proposed. One of the most accredited theories is the amyloid cascade hypothesis [1], which identifies as the main cause of AD progression the misfolding and the extracellular aggregation of Amyloid-β (Aβ) peptides from the cleavage of amyloid precursor protein (APP), as well as the intracellular deposition of the misfolded tau protein in neurofibrillary tangles. The Aβ aggregation leads to the formation of oligomeric toxic species, which can further aggregate in more ordered structures, called fibrils or fibres [2], up to the formation of extracellular senile plaques [3,4]. Among different lengths of Aβ peptides, senile aggregates are mostly made by the Aβ_40_ fibrils, but the most toxic species are the Aβ_42_ ones, due to their intrinsic tendency to self-assembly [5]. The stability of these structures is strongly linked with the progression and severity of the disease, and in the last years, many efforts have been made to characterize the molecular stability of amyloid aggregates [6,7,8,9,10,11,12,13,14].

In the past, several strategies have been developed to reduce or prevent Aβ production and to destabilize Aβ aggregates, including immunotherapeutic vaccines [15,16], antibodies [17,18], peptides [19,20], nanoparticles [21,22,23] and compounds targeting Aβ secretases [24,25] and Aβ aggregation [26,27,28,29,30,31]. However, some of these approaches have shown serious side effects [32,33] and poor permeability through the blood-brain barrier (BBB) [34]. In this context, small molecules based on natural compounds are promising inhibitors with minimal side effects and increased BBB permeability [35]. Several in vitro and in vivo studies have highlighted the potential therapeutic effects of natural compounds against neurodegenerative diseases, including AD [36,37,38,39,40,41,42]. However, their effects affect several aspects associated with AD, and their molecular mechanism of action is still not clear, consequently reducing the percentage of compounds at the clinical trial stage [3]. Hence, a deep characterization of the molecular structure of amyloid aggregates and their interactions with promising compounds, such as natural ones, is of primary importance for the design of new efficient strategies against neurodegenerative diseases [43].

In this regard, computational methods, such as molecular dynamics (MD) simulations, thanks to a detailed molecular resolution, could represent a powerful tool to shed light on the molecular mechanisms characterizing physiological and pathological phenomena [44]. Thanks to these methods, several small molecules have been proposed as amyloid antiaggregating agents [45]. A promising inhibitor, referred to as wgx-50, has shown destabilizing effects against Aβ fibrils and inhibition of neural apoptosis and apoptotic gene expression [46,47,48]. Moreover, polyproline chains have demonstrated a conversion mechanism of the Aβ secondary structure from beta-sheet to random coil, highlighting the stabilizing role of amyloid C-terminal residues [49]. Sharma et al. have evaluated the stoichiometric ratio of caffeine to the Aβ-derived switch-peptide by a combination of experimental and computational approaches, observing the peptide disaggregation when the caffeine stoichiometric is ten times higher than the peptide one [50]. Furthermore, curcumin-like compounds have been synthetized and tested on Aβ_40_ showing two binding sites, one in the 17–21 region and one near the Met35 [51], which have been previously observed by experimental and computational works [52,53]. Finally, the interactions between homotaurine, scyllo-inositol and the Aβ_42_ peptide at the monomer level have been extensively investigated by very long replica exchange MD with solute tempering simulations of 160 µs for each system, showing conformational changes of the Aβ_42_ monomer through a nonspecific binding mechanism [31].

In this context, it is worth mentioning that molecular modelling investigations have focused mostly on a specific Aβ_42_ polymorphic structure, called U-shaped fibril [54]. However, the Aβ_42_ may arrange also in other polymorphic structures, such as the S-shaped structural rearrangement [55]. Interestingly, recent works have indicated that the S-shaped structure is characterized by superior conformational and mechanical stability with respect to the U-shaped one, suggesting a correlation between structural stability and toxicity [11,12,56].

Based on the above-mentioned premises, this research work investigates the binding and action mechanisms of 57 natural compounds targeting the S-shape Aβ_42_ fibril by ensemble docking and molecular dynamics (MD) simulations. Ligands were selected starting from previous literature, and in particular in vivo or in vitro data showing their effect on the onset and progression of several neurological diseases, including AD [3,37,38,39,40]. Our results revealed the ligand’s specific mechanisms of action on amyloid aggregates. More in detail, ligands can be distinguished based on their ability to disrupt or preserve the ordered conformational structure of the amyloid fibril.

## 2. Results

**Figure 1:**
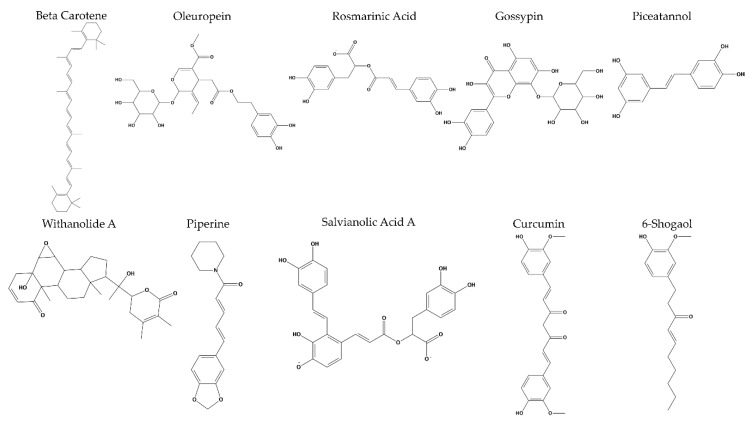
The ten best natural compounds that exhibited the lowest MM–GBSA binding energies for the selected S-shape amyloid fibril.

We first performed long MD simulations of the compound-free Aβ_42_ pentamer in order to obtain a representative structure for the docking protocol. The conformational stability of the three independent replicas of Aβ_42_ was demonstrated by the RMSD in the Supporting Information (Figure S1). The MM–GBSA binding energy estimation of the 57 ligand–receptor complexes was computed during 1 ns of MD simulation, as previously done in the literature [57], and the results are reported in the Supporting Information (Table S1). The best ten compounds, characterized by the lowest values of binding energy, were selected (Figure 1) and further characterized by long MD simulations of 150 ns. Docking poses of the best compounds and their interaction maps are reported in the Supporting Information (Figures S3 and S4). See the Materials and Methods section for further details.

**Figure 2:**
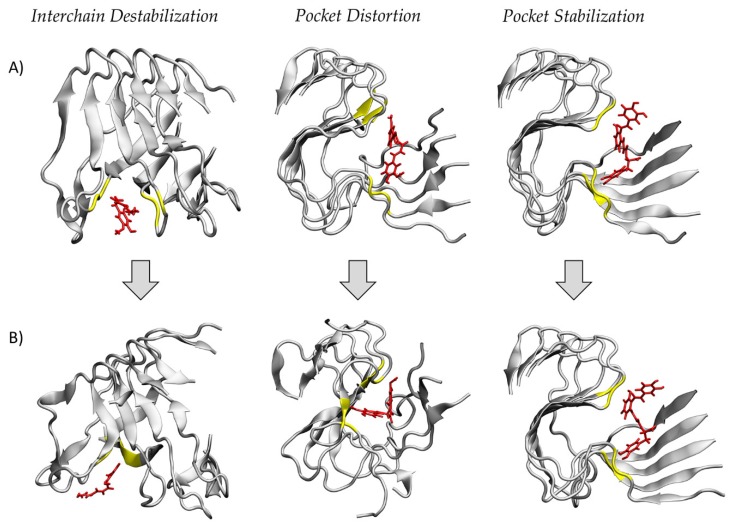
Representative snapshots of the three different mechanisms of action of selected natural compounds: interchain destabilization, pocket distortion and pocket stabilization. For each mechanism the (**A**) starting configurations after the docking protocol and the (**B**) final structures after 150 ns of molecular dynamics (MD) simulation are shown. The ligands are represented in red, while the amyloid fibrils and their residues within 0.35 nm from the ligand are represented in grey and yellow, respectively.

The MD simulations have pointed out three different mechanisms of action on the structural stability of the amyloid fibril. More in detail, (I) 6-shogaol and oleuropein were able to dock between adjacent receptor chains, inducing a considerable destabilizing effect on the whole protein; (II) curcumin, gossypin and piceatannol disrupted the ordered structure of the amyloid fibril after binding into a pocket formed by the protein S-shape; and (III) the remaining compounds, i.e., salvianolic acid A, beta-carotene, piperine, rosmarinic acid and withanolide A, did not result in remarkable protein conformational changes, thus suggesting a binding pocket stabilization. Representative snapshots of the three different mechanisms of action are reported in Figure 2.

**Figure 3:**
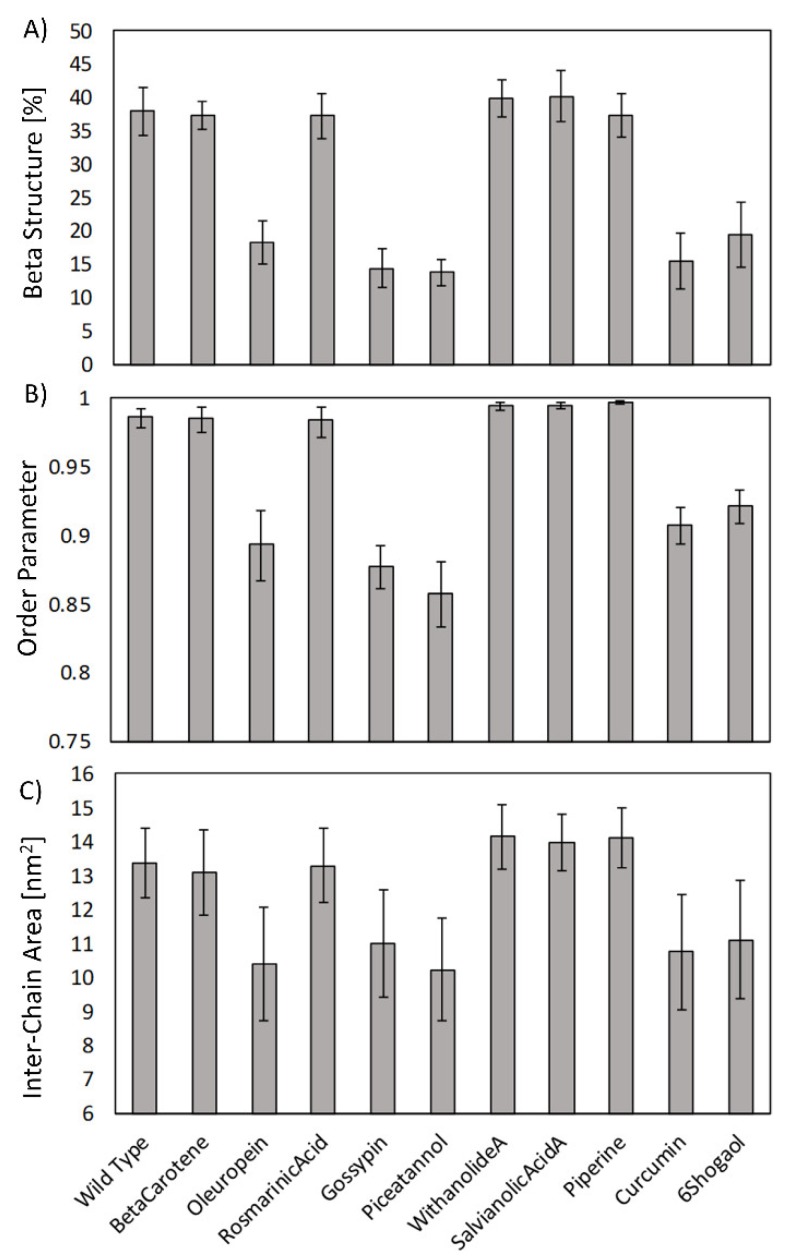
(**A**) Beta-sheet structure probability, (**B**) order parameter and (**C**) inter-chain interaction area for the wild type amyloid fibrils and all the receptor–ligand complexes.

In order to quantify the effects of the investigated compounds on the structural order of amyloid aggregates, three parameters were calculated on the last 25 ns of the MD simulations: (1) the beta-sheet structure probability; (2) the order parameter, calculated as described in Materials and Methods; and (3) the inter-chain interaction area, which measured the average contact surface between adjacent protein chains with a distance cut-off of 0.35 nm. All the above-mentioned analyses have been computed for the wild type protein, i.e., the ligand-free structure, and for all ligand–receptor complexes (Figure 3).

In Figure 3A, the beta-sheet probability is shown. The Aβ_42_ wild type was characterized by a beta structure percentage of 37.9% ± 3.6% and each compound exhibited different effects on the protein conformational stability. Among the investigated compounds, only oleuropein, gossypin, piceatannol, curcumin and 6-shogaol proved to remarkably reduce the amyloid beta structure content. Similar trends were obtained for both the geometric order parameter and the inter-chain interaction area (Figure 3B,C). Therefore, oleuropein, gossypin, piceatannol, curcumin and 6-shogaol showed destabilizing effects by inducing a reduction in terms of (i) the protein beta sheet structure content, (ii) the fibril order (quantified by the estimated order parameter) and (iii) the inter-chain interaction surface. On the above-mentioned three indicators, all the other investigated compounds demonstrated a negligible impact on the amyloid structure.

**Figure 4:**
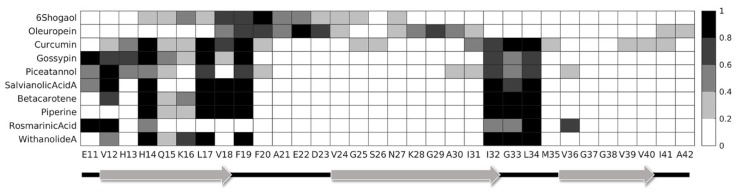
Contact probability between the selected natural compounds and the amyloid residues during the MD simulations.

From the visual inspection of the ligand–receptor binding mechanisms, it was evident that different compounds interacted with different areas of the protein, making contact with distinct residues. Hence, to better quantify these differences, the ligand–receptor contact probability was evaluated. For each MD trajectory frame, the distance between all Aβ_42_ residues and the considered compound was calculated, and the contact was counted if this distance was below a cut-off of 0.35 nm. The contact probability was then defined as the total number of contacts divided by the total number of simulation frames. The contact probability between each ligand and protein residue is reported in Figure 4. It should be noted that we have not distinguished between the same residues of different protein chains, and therefore the obtained heatmap underlines the probability of interaction of a specific residue with the considered compound. This method was chosen since the original structure of the fibrillar aggregate is formed by the repetition of identical laterally bound chains. Therefore, the only noteworthy information is the residue type involved in the interaction and not the chain that it belongs to. Furthermore, it is worth noticing that 6-shogaol detached from its binding site during the simulation. Therefore, its contact probability was estimated only for the frames in which the compound was effectively docked into the binding site. In detail, the contact probability map identified two main binding areas: residues E11–F19 and residues I32–L34. Residues mainly involved in the binding process are mostly non-polar (50%) and basic (25%), suggesting that these properties are of primary importance for an effective ligand binding. Moreover, it is important to mention that 6-shogaol and oleuropein interacted less with the above-mentioned residues, since they were docked between adjacent protein chains. In particular, 6-shogaol was shown to mostly interact with the H14–G25 region, whereas oleuropein was found mainly bound to the V18–V24 and N27–I31 ones. The other compounds, instead, were buried into the binding pocket identified by the amyloid fibril S-shape and interacted with similar residues. However, rosmarinic acid expressed a slightly different behaviour, mostly interacting with the chain edge and showing a tendency to not penetrate into the binding pocket.

**Figure 5:**
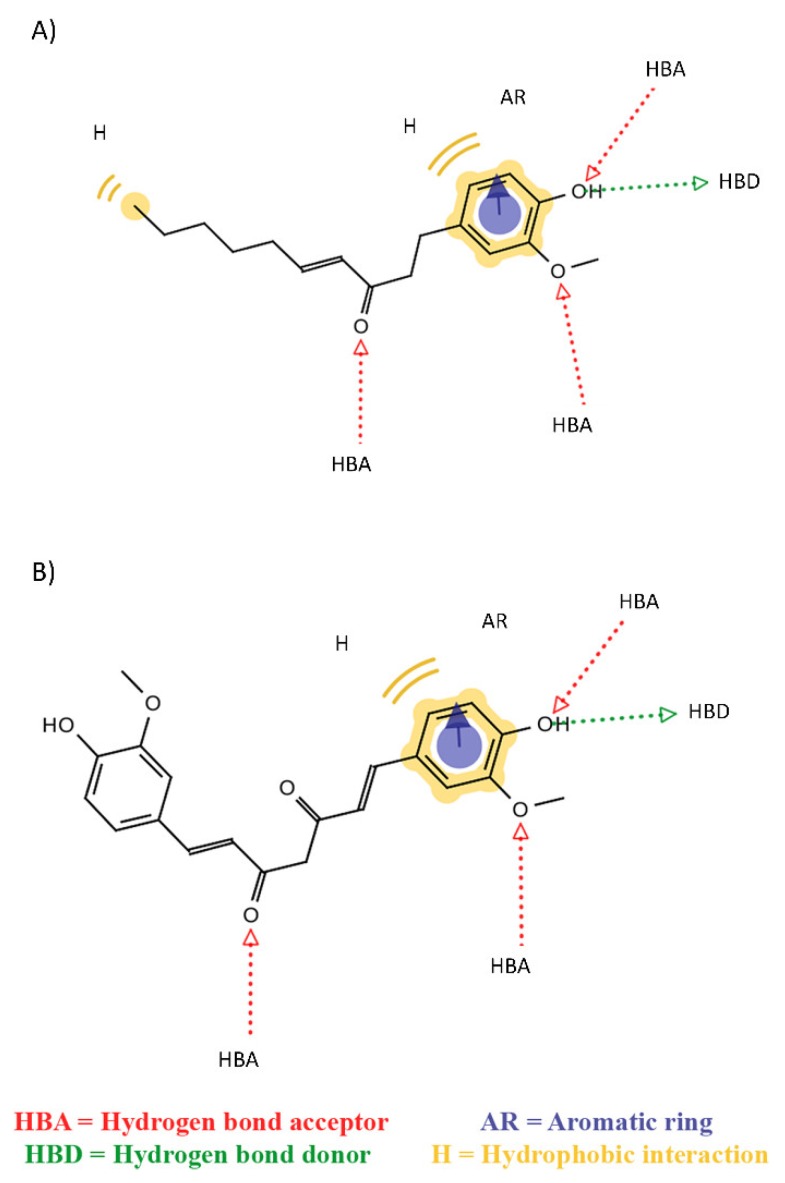
Pharmacophore model based on shared features between (**A**) 6-shogaol and oleuropein and (**B**) curcumin, gossypin and piceatannol. HBA identifies a hydrogen bond acceptor, HBD a hydrogen bond donor, AR an aromatic ring and H a hydrophobic interaction.

In order to better characterize ligand properties, which are at the basis of their mechanisms, pharmacophore modelling was performed using LigandScout software [58]. This method allowed the definition of shared features among the properties of a series of compounds. More in detail, Figure 5A represents the common features between 6-shogaol and oleuropein, which were able to dock between adjacent chains (mechanism I). Otherwise, Figure 5B shows the common features between the ligands that led to the pocket distortion (mechanism II). All destabilizing compounds share six common features, i.e., three H bond acceptors (HBA), one H bond donor (HBD), one aromatic ring (AR) and one hydrophobic interaction (H). It is worth mentioning that the first shared features model was characterized by one more hydrophobic feature than the second one. Hence, this property can probably be related to the ability of 6-shogaol and oleuropein to interpose between protein chains. Moreover, adding any one of the compounds that exhibited lower or no destabilizing effects (mechanism III), the shared pharmacophore model lost at least one “H bond acceptor” feature. For this reason, this characteristic seems crucial in the definition of the ligand destabilizing activity on the protein conformation.

## 3. Discussion

Among several neurological disorders, AD is one of the most common forms of dementia. Even though the causes of the Alzheimer’s onset and progression are still under debate, according to the amyloid cascade hypothesis [1], the amyloidogenic process that leads to the formation of extracellular aggregates of Aβ peptides is considered one of the main markers of Alzheimer’s occurrence and severity. Until now, only two strategies are used to provide symptomatic relief to AD patients: acetylcholinesterase inhibitors, to maintain the level of acetylcholine in the brain, and N-methyl-D-aspartate receptor antagonists, to prevent excitotoxicity [59]. Unfortunately, serious side effects and poor effectiveness in some phases of the disease have been detected [40,60,61]. Between different lengths of ordered and disordered amyloid peptides, the Aβ_42_ fibril is known to be the most toxic due to its tendency to self-assembly into ordered structures [5]. Moreover, this structure presents two different structural rearrangements, S-shaped and the U-shaped [54,55]. In the last years, several studies have investigated the different behaviour of these polymorphisms and the S-shaped form has demonstrated greater conformational and mechanical stability than the U-shaped form [11,12,56]. Therefore, the S-shaped structure represents the primary target for pharmacological treatments, aimed to reduce the amyloidogenic process and interfere with the amyloid aggregates’ stability. In this context, the search for destabilizers of Aβ fibrils may provide fruitful insights in the open research for treatments targeting AD. Natural compounds have shown promising effects, proving to be effective in many in vitro and in vivo studies with minimal side effects and increased blood brain barrier permeability [35]. However, the molecular mechanism of action of these compounds is still unclear and several computational studies have tried to characterize their effects on different amyloid aggregates [31,43,45,46,47,49,50,51,52,53]. In this work, a combination of ensemble docking and MD simulations has been applied to evaluate the influence of 57 promising compounds on preformed S-shape Aβ_42_ fibrils [3,37,38,39,40]. We identify three different mechanisms of action for the best ten natural compounds: (I) inter-chain destabilization, (II) pocket distortion and (III) pocket stabilization. In particular, 6-shogaol and oleuropein (mechanism I) are able to disrupt the protein ordered structure docking between adjacent fibril chains; curcumin, gossypin and piceatannol (mechanism II) dock into a binding pocket identified by the amyloid S-shape, affecting the whole protein conformation; the other ligands (mechanism III), instead, preserve or slightly influence the conformational state of the amyloid fibril. In this way, we find out that only 6-shogaol, oleuropein, curcumin, gossypin and piceatannol appreciably affect the protein stability, reducing the percentual content of beta sheets, the order parameter value and the inter-chain interaction area if compared to the wild type structure. It is worth remarking that the ligands belonging to mechanisms I and II have demonstrated similar conformational effects on the amyloid fibril, inducing similar reductions of beta-sheet structure content, order parameter and inter-chain interaction area. Therefore, all the identified destabilizing compounds, i.e., 6-shogaol, curcumin, gossypin, oleuropein and piceatannol, are underlined for further investigations. Moreover, compound shared features may be used for determining a pharmacophore model to rationally design novel compounds, hopefully characterized by a more effective destabilizing strength on Aβ toxic assemblies. To remark on the importance of the selected compounds’ chemical features, it is worth mentioning that brazilin, a modulator of the amyloid fibril conformation [62], shares five common features with the here characterized destabilizing ligands belonging to classes I and II (Figure S5).

The remaining compounds seem to stabilize the binding pocket, maintaining an ordered structure of the amyloid aggregate. Concerning previous literature [31,48,51,63], the present research expresses some aspects of novelty. In particular, this work considers the S-shaped polymorphism as the ligand target. Previous computational works have mostly studied a different amyloid polymorphism, namely the U-shaped one [47,48,51], which might be less stable than the S-shaped one [11,12]. Moreover, this work also provides a comprehensive comparative investigation on a considerable number of natural inhibitors, investigating their binding and action mechanisms.

Most of the selected compounds have shown antioxidant and anti-inflammatory properties in vivo [3,64,65,66,67]. Recent studies have remarked the destabilization action of curcumin on Aβ_40_ and Aβ_42_ [68], also with advanced amyloid accumulation [63,69]. Furthermore, it has been observed that oleuropein acts against the formation of toxic oligomer and amyloid fibrils, favouring the formation of non-toxic aggregates and improving cognitive functions [3,65,70,71,72]. It is worth mentioning that mechanism-III compounds have proven beneficial effects on AD onset and progression. Therefore, their mechanism of action probably alters the fibril structure in a different stage of the pathology or mostly affects other important factors of the disease, including the oxidative stress, the tau hyperphosphorylation, the α-secretase expression and the β-secretase activity.

Most ligands, except for 6-shogaol and oleuropein, interact with common residues in two main binding areas, identified by residues E11–F19 and I32–L34. Therefore, these residues seem crucial for the definition of the binding pocket and for the effective binding of the investigated compounds. Similar regions have been identified by previous studies about curcumin-like compounds in a complex with the U-shape polymorphism [51,52]. Moreover, in vitro studies have shown the key role of F19 and F20 for efficient Aβ_42_ polymerization [73]. Finally, the pharmacophore modelling highlights common features between the destabilizing compounds, outlining the presence of an additional hydrophobic characteristic for the mechanism-I compounds. This feature could probably be related to the ability of 6-shogaol and oleuropein to fit between protein chains. All ligands share aromatic characteristics that have been seen to be important for interacting with amyloidogenic aggregates [74]. Moreover, the presence of three H-bond acceptor features is common to all the destabilizing compounds, but not for the other ones, suggesting that this could be an important characteristic for an effective binding to the amyloid fibril and for the activity of the investigated chemicals.

As a limit of the present research, it is worth mentioning that molecular simulations employed in this work cannot represent the entire amyloidogenic process and the present study is focused on the action of natural compounds on preformed amyloid fibrils. Only experimental studies may access the proper time and length scales to correctly describe the whole fibrillogenic process. However, the present research represents a meaningful comparative investigation with atomic resolution, which helps the ligand screening workflow by elucidating binding and action mechanisms of a considerable number of existing natural compounds already considered in the AD research field. Future developments might consider combinations of different compounds, as well as the effect of different compound concentrations to better clarify potential cooperative or competitive mechanisms.

## 4. Materials and Methods

### 4.1. Molecular Dynamics Setup

The atomistic structure of the S-shape Aβ_42_ fibril was obtained from solid-state NMR (PDB ID: 2MXU [54]). Only five of the twelve chains of Aβ_42_ were extracted as previously done in the literature [11]. Water molecules were added to a periodic cubic box with sides of 8 nm. The total system charge was neutralized adding Na^+^ and Cl^−^ ions at a concentration of 150 mM. The AMBER99-ILDN force-field [75] and TIP3P model [76] were employed to define protein and water molecule topologies, respectively. A time step of 2 fs was used together with the LINCS constraints algorithm [77]. All subsequent operations were performed three times obtaining three different replicas in order to increase the statistics of the MD data. The systems were minimized using the steepest descent method and then simulated with position restraints on protein heavy atoms for 200 ps in NVT ensemble using the V-rescale coupling method [78] to maintain a temperature of 300 K. We further simulated the system with the above-described position restraints in NPT ensemble for 400 ps using the V-rescale thermostat [78] and isotropic Berendsen barostat [79] to maintain temperature (300 K) and pressure (1 bar), respectively. Finally, MD simulations were performed without any restraints for 200 ns under NPT ensample, using the V-rescale [78] and Parrinello-Rahman [80] coupling methods. The short-ranged Van der Waals (VDW) interactions were cut off after 1 nm and long-ranged electrostatic interactions were calculated using the Particle Mesh Ewald (PME) method [81]. All simulations were carried out by GROMACS 2018 software package [82], while the Visual Molecular Dynamics (VMD) package was employed for the visual inspection of the simulated systems [83].

### 4.2. Molecular Docking Protocol

For each of the above-mentioned replicas, a cluster analysis was performed during the last 50 ns using linkage method [82] and a RMSD cut-off of 0.1 nm, as done previously in the literature [84]. The centroid of the most populated cluster for each replica was assumed as the starting receptor configuration (see Figure S2).

Structures of the 57 investigated ligands were downloaded from the PubChem database [85] and their protonation state was computed using Molecular Operating Environment software (MOE). The complete list of chosen natural compounds with their physiological charge was reported in the Supporting Information (Table S1). Then, ligand topologies were obtained with Antechamber [86,87] using the General Amber Force Field (GAFF) [86] and AM1-BCC charge method [88], as applied in previous studies [48,89,90,91,92].

Molecular docking was carried out by AutoDock Vina [93] using 64 as exhaustiveness and a search box of 9 nm x 9 nm x 12 nm, located at the centre of the protein and able to cover the entire protein surface. Each ligand was docked to the three different centroid configurations and only the best mode in terms of Vina binding affinity was selected, obtaining 57 receptor–ligand complexes.

### 4.3. Binding Energy Estimation and Protein-Compounds Conformational Dynamics

Each receptor–ligand complex system was followed by solvation, neutralization, energy minimization, position-restrained MD and a short MD production of 1 ns, with the same setup described in the molecular dynamics setup section. Then, the receptor–ligand binding energy was estimated by the MM–GBSA method [94] using parameters from previous literature [57,95,96].

The ten best ligands in terms of binding energy were further characterized by a MD simulation of 150 ns, in order to highlight the conformational changes of the amyloid fibril induced by the presence of natural compounds. Finally, the three starting receptor configurations were simulated using the above-mentioned protocol for 150 ns in order to compare the structural effects in the absence of ligands. Three replicas for each system were performed to check the reliability of the results. A simulations summary is reported in the Supporting Information (Table S2).

### 4.4. Order Parameter

The Aβ_42_ fibrils were characterized by a regular shape, repeated in each chain. In order to estimate the structural order of the pentamer, an order parameter (ordP) was calculated similarly to previous works [11,13,14,97]:

(1) $$ordP={〈\frac{\sum_{c=1}^{Nc}\left(\sum_{n=1}^{Nr}\left(\vec{CoM_{0}-C\alpha_{0}}\right)\cdot \left(\vec{CoM_{t}-C\alpha_{t}}\right)\right)}{Nr\;Nc}〉}_{t}$$

Here, the arrow represents the connecting vector of the centre of mass (CoM) position and alpha carbon (Cα) position of the n-th residue and of the c-th chain. The ordP is the dot product averaged along the observation time interval, the number of residues (NR) and the number of the chains (NC). Values of ordP close to 1 indicated an alignment close to the initial structure, i.e., aligned fibres along the fibril axis z. Values of ordP lower than 1 indicated a gradual structure distortion.

## 5. Conclusions

In this work, molecular modelling techniques were employed to screen among 57 natural compounds. Five ligands, i.e., 6-shogaol, oleuropein, curcumin, gossypin and piceatannol, showed a remarkable destabilizing activity on the Aβ_42_ S-shape polymorphism. Two different destabilizing modes of action of the inspected ligands were revealed. Finally, throughout pharmacophore modelling, the main common features of the highlighted ligands have been identified. These chemical features can be considered for further rational search/design of amyloid destabilizing agents. For greater detail, future studies may expand the database of the investigated compounds, including possible other interesting natural ligands characterized by shared chemical features with respect to those identified in this work. Moreover, further works may consider effects of ligand concentration, combinations of destabilizing compounds or the presence of different species of metal ions involved in the ligand-target binding mechanism.

## Supplementary Materials

Supplementary materials can be found at https://www.mdpi.com/1422-0067/21/6/2017/s1.

## Abbreviations

AD	Alzheimer’s Disease
BBB	Blood Brain Barrier
APP	Amyloid Precursor Protein
MM–GBSA	Molecular Mechanics–Generalized Born Surface Area
VDW	Van der Waals
PME	Particle Mesh Ewald
GAFF	General Amber Force Field
RMSD	Root Mean Square Deviations
ordP	Order Parameter
