# Supplementary material for: The Impact of Natural Compounds on S-Shaped Aβ42 Fibril: From Molecular Docking to Biophysical Characterization
Source: Int J Mol Sci. 2020 Mar 16;21(6):2017. doi: 10.3390/ijms21062017 (PMC7139307; doi:10.3390/ijms21062017)
Supplement: Supplementary file 1 [file ijms-21-02017-s001.pdf]

The Impact of Natural Compounds on S-Shaped Aβ42 Fibril: from Molecular Docking to Biophysical Characterization

Stefano Muscat [1,*], Lorenzo Pallante [2,*], Filip Stojceski [1], Andrea Danani [1], Gianvito Grasso [1] and Marco Agostino Deriu [2,]**

[1] Dalle Molle Institute for Artificial Intelligence (IDSIA), University of Italian Switzerland (USI), University of Applied Science and Art of Southern Switzerland (SUPSI), Manno, Switzerland.
[2] PolitoBIOMed Lab, Department of Mechanical and Aerospace Engineering. Politecnico di Torino, Italy.

*Stefano Muscat and Lorenzo Pallante contributed equally to this study.
**Correspondence: marco.deriu@polito.it

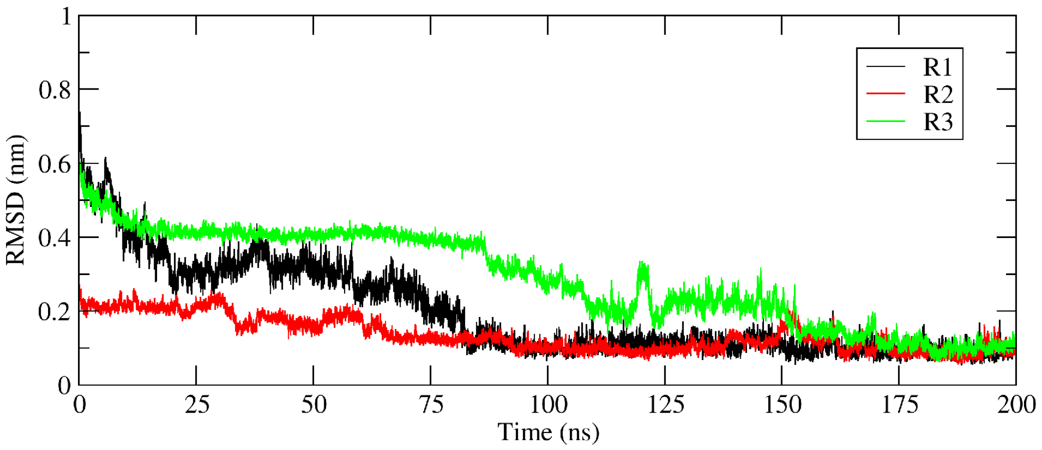

Figure S1. RMSD of the five chains of the 2MXU about the average structure during the last 25 ns. The first replica is represented in black, the second one in red and the third one in green. All systems reach the structural equilibrium.

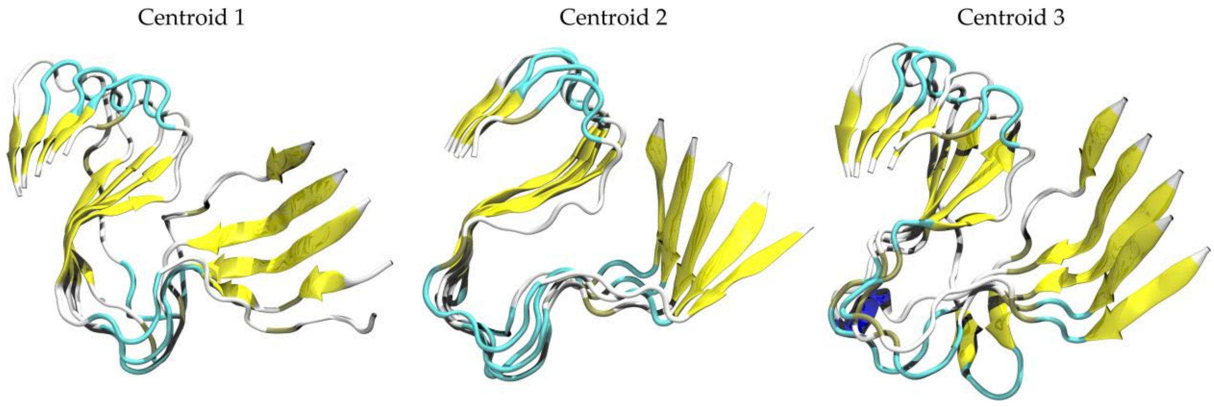

Figure S2. Centroids of the most populated cluster for each independent replica. Each configuration is obtained by a cluster analysis on the last 50 ns of MD simulations, using linkage method and a RMSD cut-off of 0.1nm.

Table S1. List of compounds with their relative binding energy and charge.

Compound	Binding Energy (kcal/mol)	Charge
Beta Carotene	-55.81	0
Oleuropein	-42.63	-1
Rosmarinic Acid	-42.61	-1
Gossypin	-40.97	-1
Piceatannol	-39.75	1
Withanolide A	-39.02	0
Salvianolic Acid A	-37.78	-1
Piperine	-35.75	0
Curcumin	-35.67	0
6-Shogaol	-34.57	-1
EGCG	-34.31	0
Myricetin	-34.28	-1
Viniferin	-34.27	-2

Epicatechin	-33.58	-1
Fisetin	-33.57	0
Diosgenin	-33.50	-1
Rutin	-33.42	-1
Asiatic Acid	-32.97	-1
Puerarin	-32.76	0
Berberine	-32.44	0
Resveratrol	-31.87	0
α-Linolenic Acid	-30.94	0
Retinal	-28.90	1
ScylloInositol	-28.54	0
Vitamine D	-28.08	-1
Rhodosin	-27.85	-1
Retinol	-27.19	0
DHA	-27.18	0
Retinoic Acid	-26.90	-2
Naringin	-26.89	-1
LTheanine	-26.61	0
Caffeic Acid	-26.41	0
Honokiol	-26.17	0
Ellagic	-26.11	0
Hydroxytyrosol	-24.82	0
Vitamine E	-24.16	0
Apigenin	-23.36	0
Quercitin	-22.33	0
NDGA	-22.01	0
Osthole	-21.97	-1
Tetracycline	-20.96	0
Baicalein	-20.91	0
Melatonin	-20.71	-1
Kaempferol	-19.98	0
Oleocanthal	-17.74	0
Naringenin	-16.60	-1
Ferulic Acid	-16.19	0
Homotaurine	-15.04	-2
Caffeine	-12.89	0
Morin	-12.50	-1
Vanillic Acid	-11.01	-1
Lipoic Acid	-9.98	0
Huperizne A	-9.53	-1
EPA	-9.19	0
Vitamine C	-7.52	0
Glycine Betaine	-0.29	0
Gallic Acid	-0.19	-1

Table S2. Summary of the simulated systems.

System	N. Replicas	Simulation Time [ns]
Configuration 1 – No Ligand	3	150
Configuration 2 – No Ligand	3	150
Configuration 3 – No Ligand	3	150
Beta Carotene	3	150
Oleuropein	3	150
Rosmarinic Acid	3	150
Gossypin	3	150
Piceatannol	3	150
Withanolide A	3	150
Salvianolic Acid A	3	150
Piperine	3	150
Curcumin	3	150
6-Shogaol	3	150

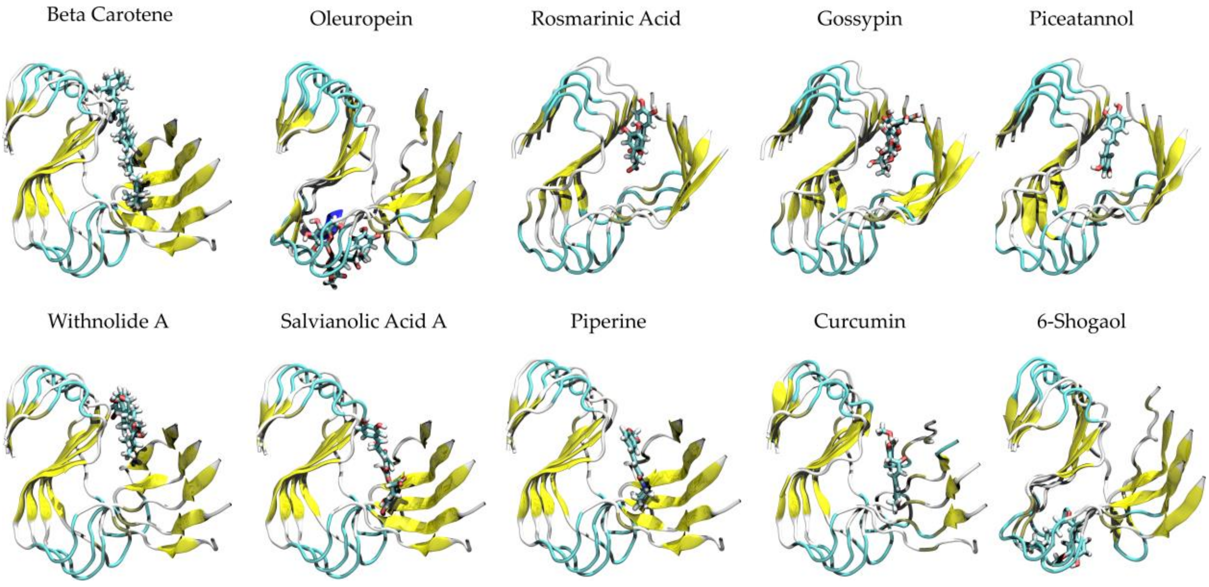

Figure S3. Docking poses of the best ten compounds on the amyloid fibril.

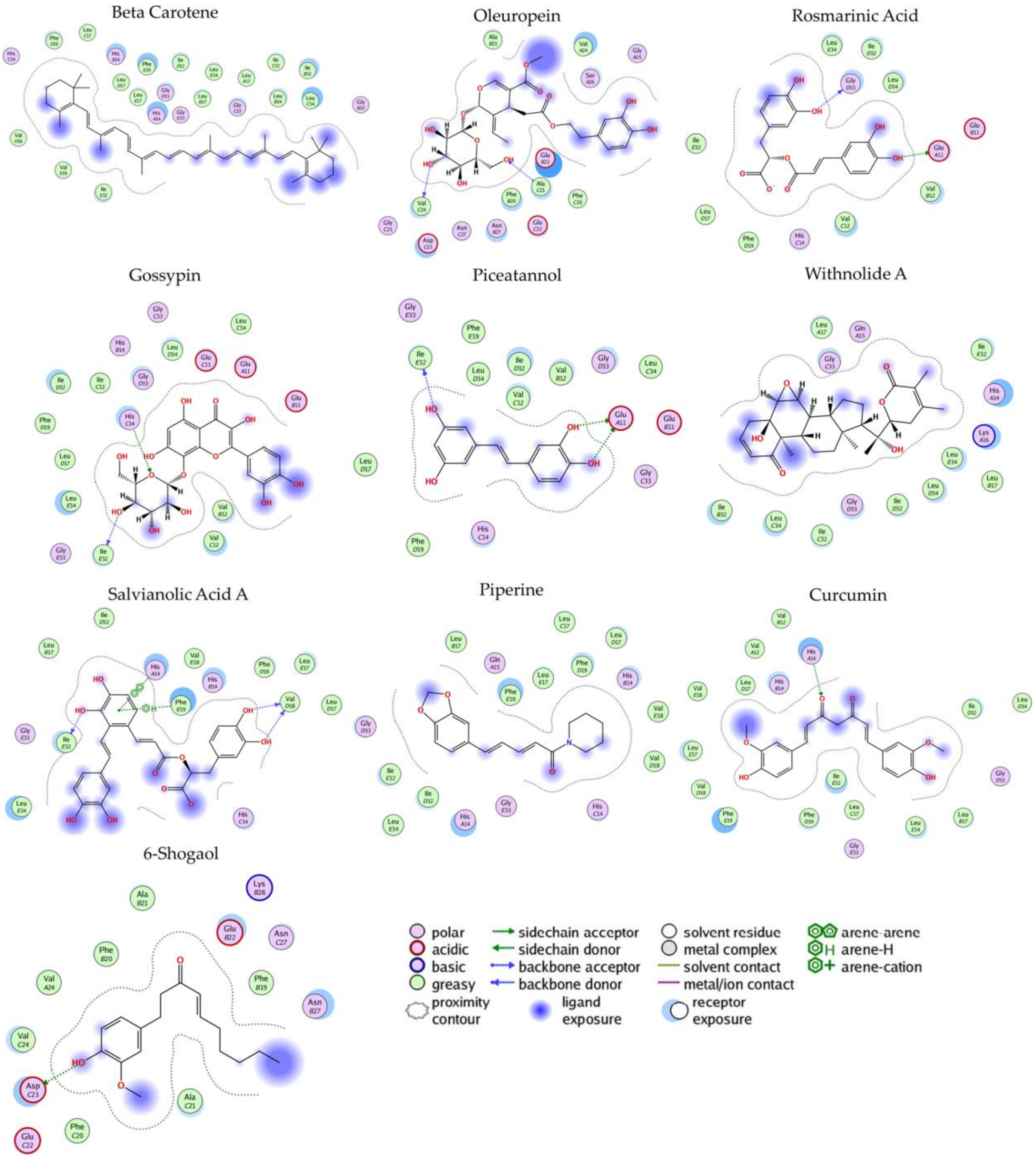

Figure S4. Ligand interactions maps for the best ten investigated natural compounds.

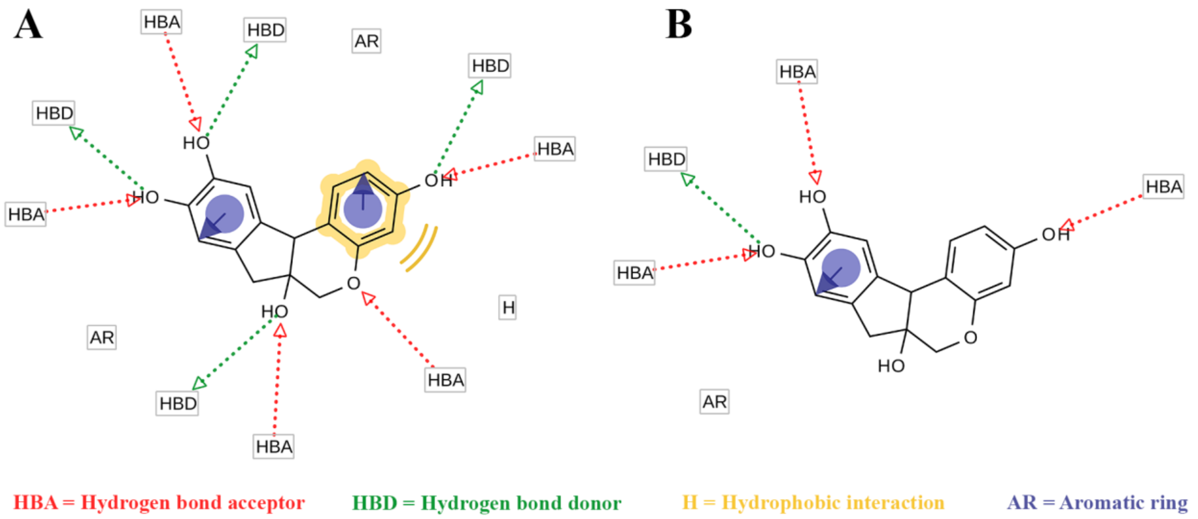

Figure S5. (A) Brazilin ligand-based pharmacophore and (B) shared features pharmacophore between brazilin and mechanism I and II destabilizing compounds, i.e. 6-shogaol, oleuropein, curcumin, gossypin and piceatannol.
